# Pilsicainide Intoxication Presenting With Alternating Accelerated Idioventricular and Junctional Escape Rhythms During Acute Kidney Injury: A Case Report

**DOI:** 10.7759/cureus.116187

**Published:** 2026-09-13

**Authors:** Shousei Osako, Eiji Miyauchi, Naoya Oketani, Mitsuru Ohishi

**Affiliations:** 1 Cardiology, Kagoshima City Hospital, Kagoshima, JPN; 2 Cardiovascular Medicine and Hypertension, Graduate School of Medical and Dental Sciences, Kagoshima, JPN

**Keywords:** accelerated idioventricular rhythm, acute kidney injury, continuous hemodiafiltration, junctional escape rhythm, pilsicainide intoxication, sodium channel blocker, wide qrs complex

## Abstract

Pilsicainide is a class Ic sodium channel blocker that is eliminated predominantly by the kidneys and may accumulate when renal function deteriorates. An 85-year-old man receiving pilsicainide 150 mg/day developed acute kidney injury (AKI) during hospitalization for hemorrhagic colitis. He subsequently developed impaired consciousness, dyspnea, pulmonary congestion, and alternating episodes of junctional escape rhythm at 30-37 beats/min and a regular, wide QRS rhythm at 79 beats/min. The QRS duration was approximately 150 ms, and the serum pilsicainide concentration was 3.19 µg/mL. The wide QRS rhythm was considered most consistent with pilsicainide-associated accelerated idioventricular rhythm accompanied by marked intraventricular conduction delay. Pilsicainide and bisoprolol were discontinued, and continuous hemodiafiltration was initiated because of severe toxicity accompanied by AKI, hyperkalemia, and volume overload. The patient’s level of consciousness, hemodynamic status, and electrocardiographic abnormalities improved; the QRS duration narrowed to 104 ms, and the serum pilsicainide concentration decreased to 0.8 µg/mL. This case emphasizes that a relatively slow, regular, wide QRS rhythm in a patient with renal impairment should prompt a review of sodium channel blocker exposure. Medication history, renal function, serum drug concentration, and serial electrocardiograms should be integrated into the diagnostic approach when differentiating accelerated idioventricular rhythm from ventricular tachycardia.

## Introduction

Pilsicainide is a pure class Ic antiarrhythmic agent with relatively selective cardiac sodium channel-blocking activity and is used in Japan for atrial tachyarrhythmias [1]. The drug is eliminated predominantly through the kidneys, largely unchanged; consequently, renal impairment increases exposure and the risk of drug-induced proarrhythmia [1-3].

At toxic concentrations, pilsicainide markedly slows myocardial conduction and may also depress impulse formation and contractility. Reported electrocardiographic manifestations include sinus node and atrioventricular conduction disturbances, junctional escape rhythm, QRS prolongation, ventricular tachycardia, ventricular fibrillation, and torsades de pointes [3-7]. Similar wide QRS toxic syndromes occur with other class Ic sodium channel blockers, including flecainide [8].

We report a case of pilsicainide intoxication caused by acute kidney injury (AKI) superimposed on chronic kidney disease, presenting with alternating episodes of junctional escape rhythm and a regular, wide QRS rhythm considered most consistent with accelerated idioventricular rhythm - an ectopic ventricular rhythm typically characterized by wide QRS complexes with an intrinsic rate of 60 to 100 beats per minute, distinct from ventricular tachycardia - which was accompanied by marked intraventricular conduction delay. The case illustrates the importance of renal dose reassessment, cautious interpretation of relatively slow, wide QRS rhythms, and prompt correction of the factors sustaining severe toxicity.

## Case presentation

Patient information

An 85-year-old Japanese man had a history of hypertension, chronic kidney disease, and paroxysmal atrial fibrillation. His recent baseline estimated glomerular filtration rate (eGFR) was approximately 75 mL/min/1.73 m². His medications included pilsicainide hydrochloride 150 mg/day (50 mg three times daily), rivaroxaban, oral and transdermal bisoprolol, spironolactone, dapagliflozin, and furosemide. He had recently been hospitalized for hemorrhagic colitis. Subsequent volume depletion was followed by a progressive decline in renal function, with the eGFR decreasing to 26.7 mL/min/1.73 m² (Kidney Disease: Improving Global Outcomes (KDIGO) criteria Stage 2 AKI) (Figure 1) [9]. The exact onset of AKI could not be ascertained. The pilsicainide dose was not adjusted after this deterioration in renal function. The patient took the last dose after lunch on the day of emergency transport, approximately six hours prior to arrival.

**Figure 1:**
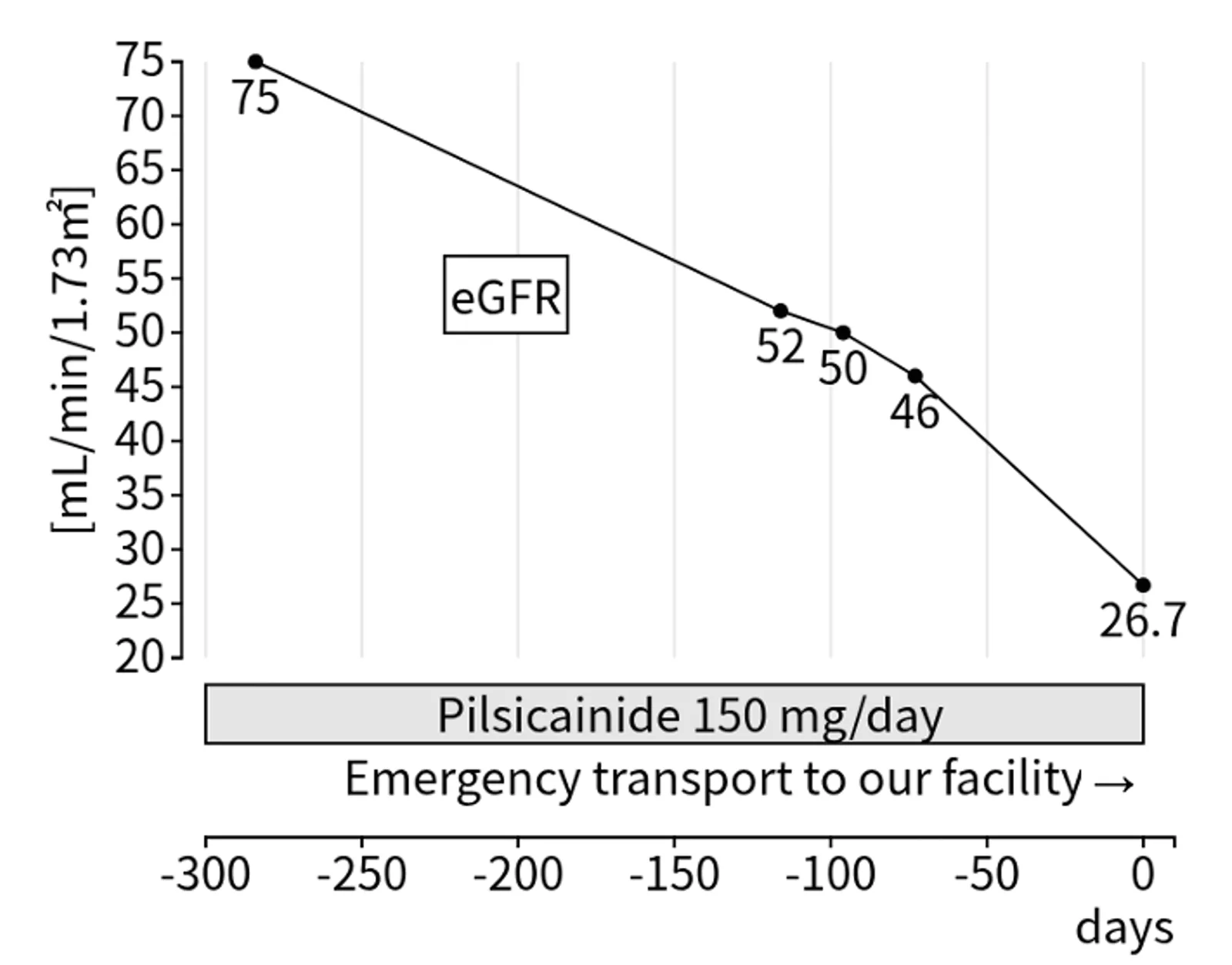
Progressive decline in renal function before admission Clinical course showing the decline in estimated glomerular filtration rate (eGFR) following hospitalization for hemorrhagic colitis, consistent with acute kidney injury superimposed on chronic kidney disease.

Clinical findings on admission

He presented to the emergency department with impaired consciousness, bradycardia, and dyspnea. His blood pressure was 156/102 mmHg, heart rate was 79 beats/min, respiratory rate was 24 breaths/min, and oxygen saturation was 94% on room air. Physical examination revealed pulmonary crackles and peripheral edema. Initial laboratory workup was significant for acute-on-chronic kidney injury (creatinine, 1.91 mg/dL), hyperkalemia (potassium, 5.6 mEq/L), and elevated B-type natriuretic peptide. Venous blood gas analysis showed a pH of 7.386 and bicarbonate of 19.8 mmol/L. Total serum calcium was 7.7 mg/dL with albumin of 2.8 g/dL (corrected calcium, 8.9 mg/dL); magnesium was not obtained on admission. Electrocardiography demonstrated two alternating rhythm patterns. The first was a regular, wide QRS rhythm at 79 beats/min, with a QRS duration of approximately 150 ms and left-axis deviation (Figure 2). P waves were not clearly identifiable. The second pattern was severe bradycardia caused by junctional escape rhythm at 30-37 beats/min (Figure 3).

**Figure 2:**
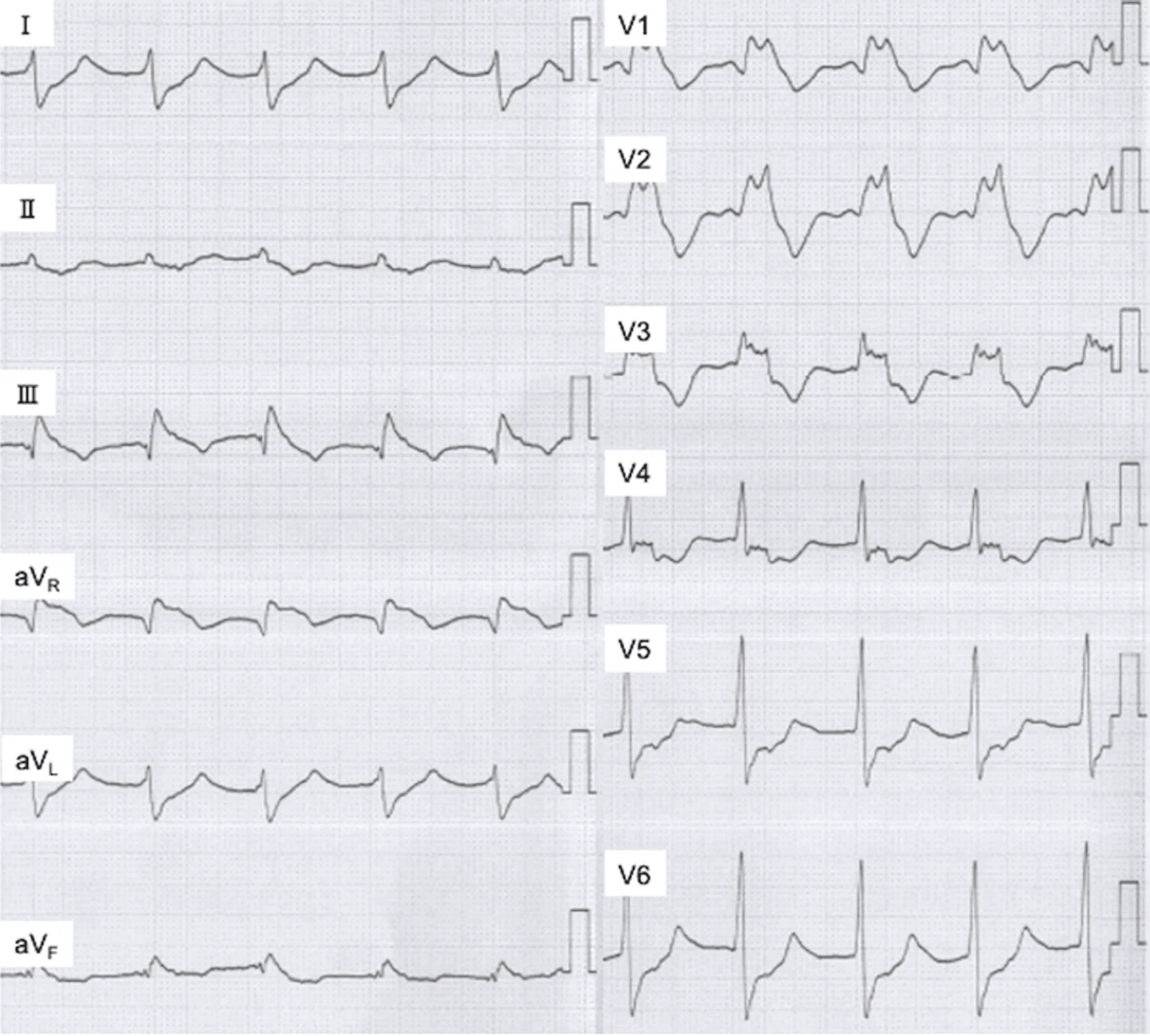
Twelve-lead electrocardiogram on admission showing regular, wide QRS rhythm most consistent with accelerated idioventricular rhythm The 12-lead electrocardiogram demonstrates a regular, wide QRS rhythm at 79 beats/min with a QRS duration of approximately 150 ms, marked intraventricular conduction delay, and absence of identifiable P waves (recorded at 25 mm/s and 10 mm/mV). A prominent terminal R wave (~4 mm) is observed in lead aVR. Given the severe pilsicainide toxicity in the setting of acute kidney injury, an alternating junctional escape rhythm, and resolution upon recovery, the rhythm was diagnosed as pilsicainide-associated accelerated idioventricular rhythm.

**Figure 3:**
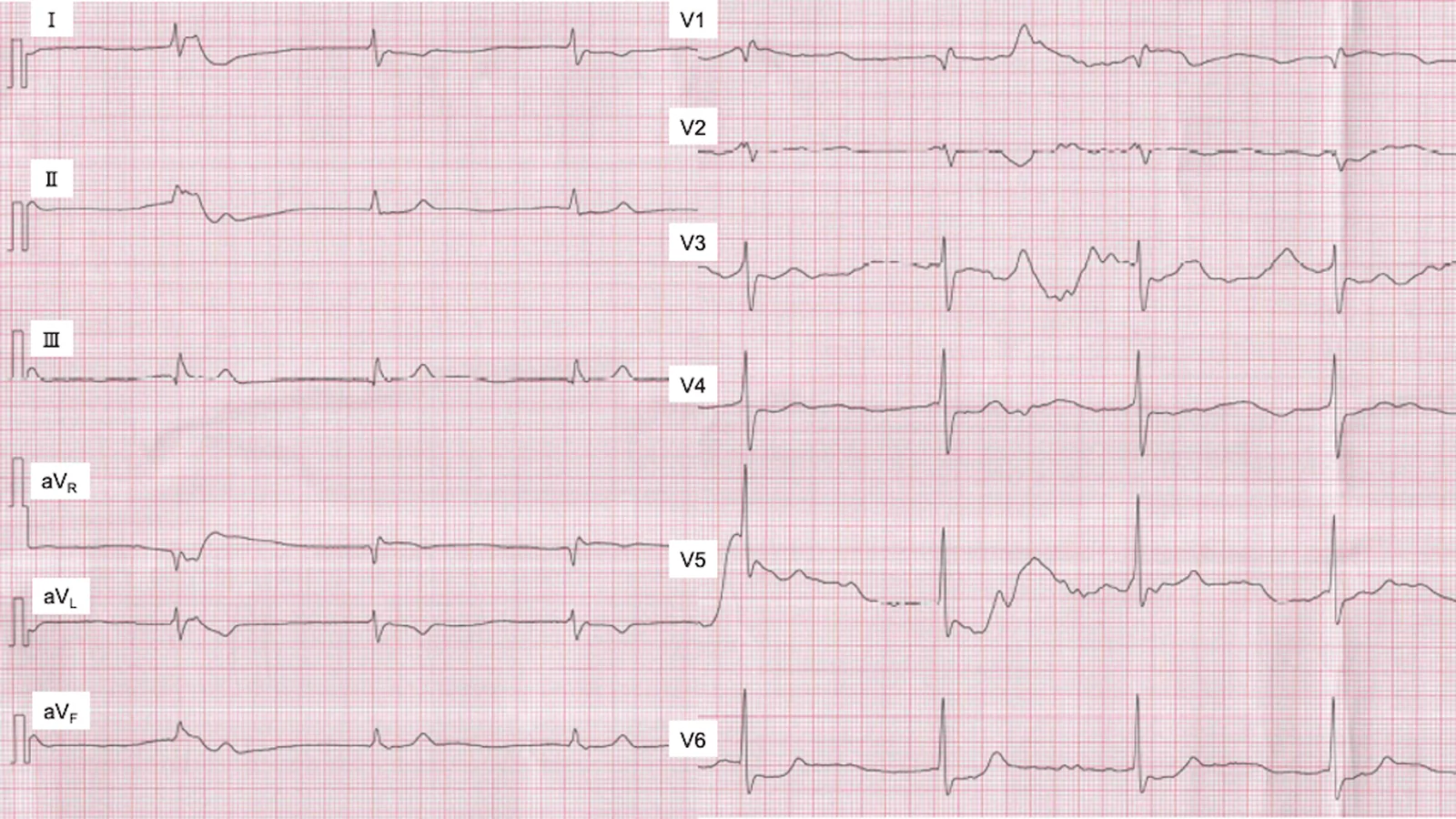
Twelve-lead electrocardiogram during severe bradycardia A junctional escape rhythm is present at approximately 37 beats/min (R-R interval approximately 1.6 s) with marked intraventricular conduction delay (paper speed 25 mm/s; calibration 10 mm/mV). Retrograde P waves are not clearly identifiable.

Diagnostic assessment

Pilsicainide toxicity was suspected because of recent deterioration in renal function while maintaining a pilsicainide dosage of 150 mg/day. The serum pilsicainide concentration measured shortly after admission was 3.19 µg/mL, markedly exceeding the conventional therapeutic range of 0.2-0.9 µg/mL and well above therapeutic concentrations reported in previous studies and case reports [2,5,7]. Transthoracic echocardiography revealed a left ventricular ejection fraction of approximately 50%, mild left atrial enlargement, and no regional wall motion abnormalities. The differential diagnosis included ventricular tachycardia, accelerated idioventricular rhythm, supraventricular rhythm with aberrant conduction, hyperkalemia-related conduction disturbance, and drug-induced intraventricular conduction delay. Accelerated idioventricular rhythm is generally defined as at least three consecutive ventricular beats occurring at a rate similar to or modestly faster than the competing supraventricular rhythm, typically around 50-120 beats/min [10]. The observed rate of 79 beats/min was compatible with accelerated idioventricular rhythm, although not diagnostic on its own. The markedly elevated serum pilsicainide concentration, AKI, alternating junctional escape rhythm, and subsequent resolution of the wide QRS rhythm during clinical and biochemical recovery made pilsicainide-associated accelerated idioventricular rhythm the most plausible diagnosis.

Therapeutic intervention

Pilsicainide and bisoprolol were discontinued. Continuous hemodiafiltration (CHDF) was initiated because of severe toxicity accompanied by AKI, hyperkalemia, and pulmonary congestion. CHDF was expected to assist in correcting electrolyte, acid-base, and volume abnormalities and might also contribute to drug elimination, although the reported extracorporeal removal of pilsicainide is limited [3-5,7]. Intravenous sodium bicarbonate was not administered because of pulmonary congestion and concern about additional sodium and volume load. Sodium bicarbonate is commonly used for QRS widening caused by other sodium channel blockers, particularly tricyclic antidepressants and flecainide, but evidence specific to pilsicainide is limited [4,11].

The patient’s level of consciousness, hemodynamic status, and electrocardiographic abnormalities improved within hours of pilsicainide withdrawal and CHDF initiation. Both the accelerated idioventricular rhythm and the junctional escape rhythm disappeared. Following the initiation of CHDF, the serum potassium level decreased from 5.6 mEq/L on day 1 to 4.9 mEq/L on day 2 and 4.3 mEq/L on day 3, thereafter stabilizing between 4.6 and 4.9 mEq/L (Figure 4). The serum pilsicainide concentration subsequently decreased from 3.19 to 0.8 µg/mL. Because drug withdrawal, renal replacement therapy, and correction of metabolic and volume abnormalities occurred concurrently, the contribution of each intervention could not be isolated. Changes in serum pilsicainide concentration and systolic blood pressure, together with the timing of CHDF and rhythm recovery, as well as serum potassium levels, are summarized in Figure 4.

**Figure 4:**
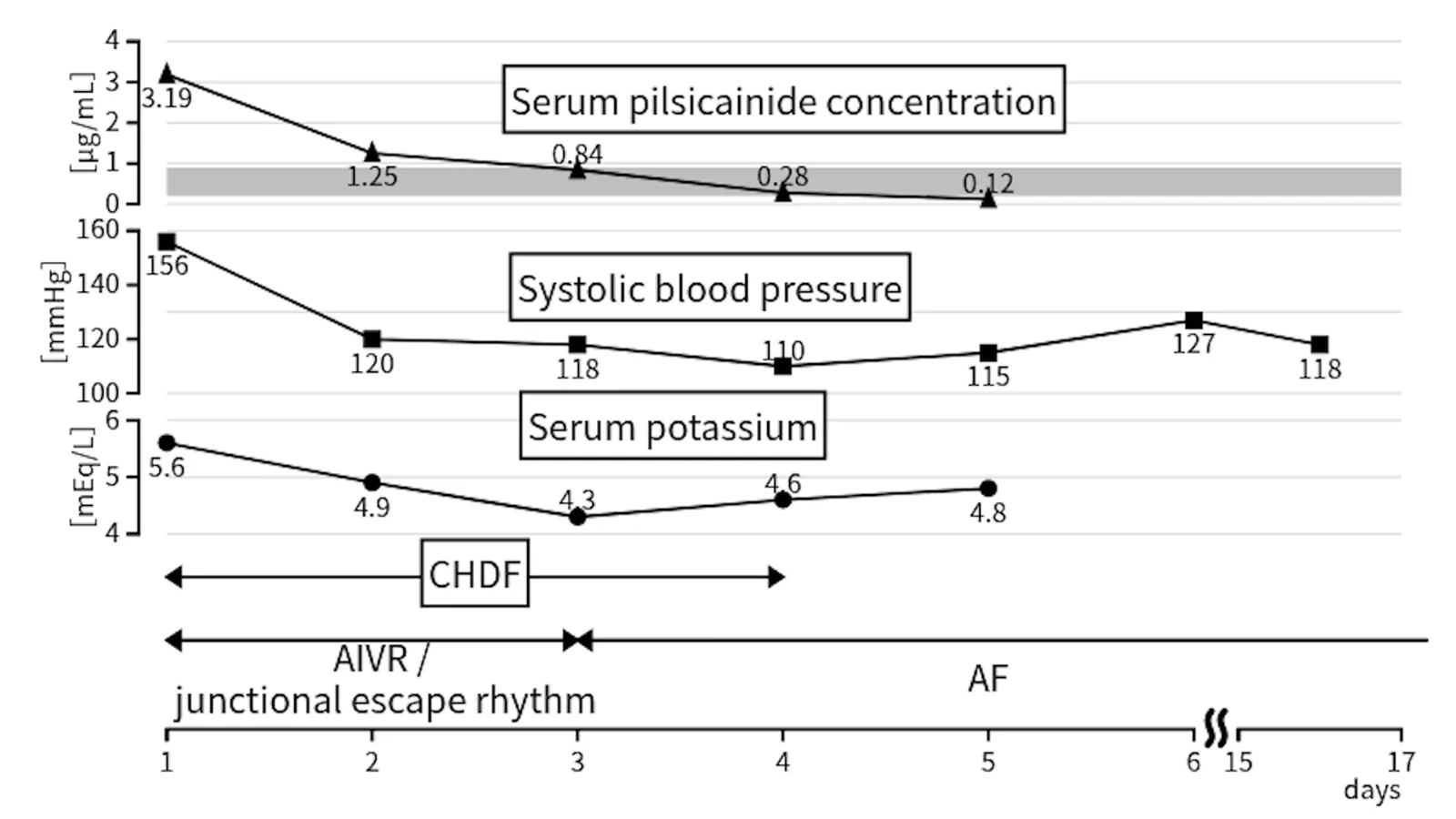
Clinical course after admission and initiation of continuous hemodiafiltration (CHDF) Serial changes in serum pilsicainide concentration and systolic blood pressure are shown, together with the duration of CHDF and the corresponding cardiac rhythms. The shaded area indicates the reported therapeutic range of serum pilsicainide concentration. The rhythm annotations show the transition from alternating accelerated idioventricular rhythm (AIVR) and junctional escape rhythm to the patient's baseline atrial fibrillation (AF). The break in the time axis between days 6 and 15 indicates the interval between hospital discharge and follow-up.

Follow-up and outcomes

Following pilsicainide withdrawal and CHDF, the rhythm returned to the patient’s baseline atrial fibrillation with a controlled ventricular response, and the QRS duration narrowed to 104 ms (Figure 5). Renal function and serum electrolyte concentrations gradually improved. He was discharged on day 9 without recurrence of the accelerated idioventricular rhythm or severe bradycardia. Pilsicainide was permanently discontinued, and rate control was subsequently maintained with low-dose beta-blocker therapy.

**Figure 5:**
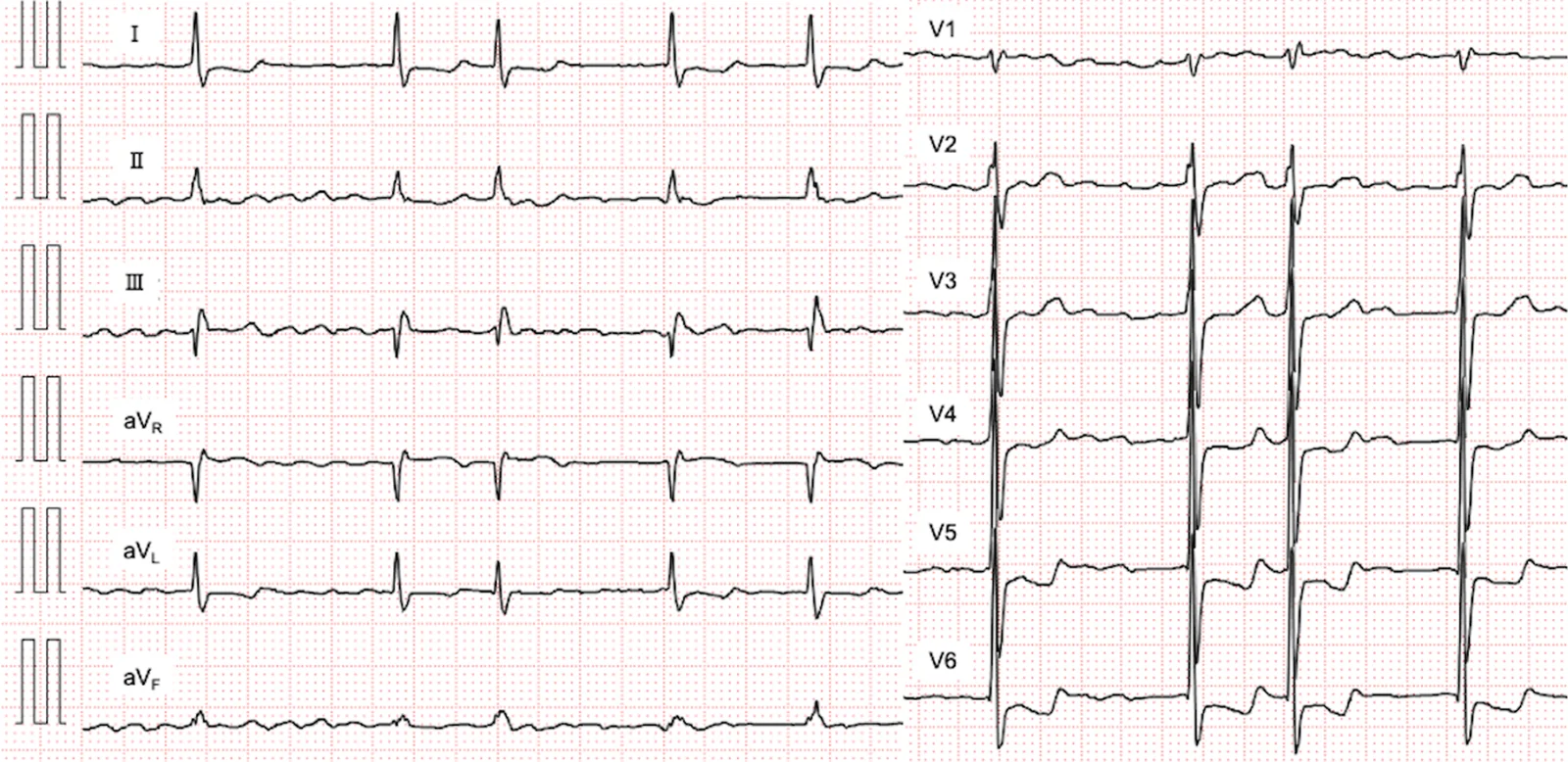
Electrocardiogram after stabilization following continuous hemodiafiltration (CHDF) The 12-lead electrocardiogram obtained on day 4 (after CHDF discontinuation) shows disappearance of accelerated idioventricular rhythm and junctional escape rhythm, with restoration of the patient's baseline atrial fibrillation. The QRS duration narrowed from approximately 150 ms on admission to 104 ms as the patient's clinical condition improved and serum pilsicainide concentration decreased. (Paper speed 25 mm/s; amplitude 10 mm/mV.)

## Discussion

Mechanism of intoxication

Pilsicainide is eliminated predominantly by the kidneys, and renal dysfunction increases exposure and prolongs elimination [1-3]. In this patient, volume depletion after hemorrhagic colitis was followed by AKI superimposed on chronic kidney disease while the full pilsicainide dose was continued. This temporal sequence provides a plausible explanation for the markedly elevated serum concentration. Pilsicainide intoxication precipitated by dehydration has been reported previously [3,5]. Concurrent oral and transdermal bisoprolol may have contributed to bradycardia through additive negative chronotropic effects; however, the relative contribution of bisoprolol cannot be determined from this case.

Electrocardiographic manifestations

A prominent terminal R wave was observed in lead aVR. In a prospective cohort of acute tricyclic antidepressant poisoning, a terminal R wave of at least 3 mm in lead aVR was associated with seizures or ventricular arrhythmias [12]. This finding reflects sodium channel blockade but has not been validated as a specific marker of pilsicainide toxicity or as a discriminator between accelerated idioventricular rhythm and ventricular tachycardia. It should not be used in isolation.

Differential diagnosis

In this case, the rate of 79 beats/min, AKI, markedly elevated serum pilsicainide concentration, alternating junctional escape rhythm, and disappearance of the rhythm during recovery collectively favored pilsicainide-associated accelerated idioventricular rhythm. Atrioventricular dissociation, capture beats, and fusion beats support ventricular tachycardia when present; however, their absence does not exclude ventricular tachycardia, and atrioventricular dissociation may also occur during accelerated idioventricular rhythm. Moreover, P waves were not clearly identifiable in this patient. The diagnosis should therefore be regarded as the most likely interpretation based on integrated clinical and serial electrocardiographic assessment, rather than as proof based on a single tracing. Because acute pharmacological treatments for hyperkalemia, such as intravenous calcium gluconate, were not administered prior to CHDF, the potential electrocardiographic overlap and synergistic effects between hyperkalemia (5.6 mEq/L) and pilsicainide toxicity could not be independently differentiated. The principal features considered in the differential diagnosis are summarized in Table 1.

**Table 1:** Findings considered in the differential diagnosis of ventricular tachycardia (VT) and accelerated idioventricular rhythm (AIVR) in the present case AV: atrioventricular; CHDF: continuous hemodiafiltration; ECG: electrocardiogram

Feature	VT	Findings in the present case favoring AIVR
Ventricular rate	Typically >100-120 beats/min, but slow VT can occur	79 beats/min, compatible with AIVR but not diagnostic
AV relationship	AV dissociation, capture beats, or fusion beats support VT when present	P waves were not clearly identifiable; the absence of AV dissociation was not used to exclude VT
QRS complex	Wide; morphology depends on ventricular origin and underlying conduction disease	Approximately 150 ms with severe drug-associated intraventricular conduction delay
Clinical context	Ischemia, myocardial scar, structural heart disease, or channelopathy may be present	Acute kidney injury with serum pilsicainide concentration of 3.19 µg/mL
Associated rhythm	Variable	Alternated with junctional escape rhythm at 30-37 beats/min
Serial course	Depends on mechanism and treatment	Disappeared during drug withdrawal, CHDF, and correction of metabolic and volume abnormalities
Overall interpretation	Diagnosis based on the ECG and clinical context	AIVR was the most likely diagnosis; ventricular origin was not established by a single criterion

Therapeutic considerations

There is no established specific antidote for pilsicainide toxicity. Management begins with immediate withdrawal of pilsicainide, correction of electrolyte and acid-base abnormalities, respiratory and hemodynamic support, and treatment of unstable arrhythmias. Sodium bicarbonate is widely used for cardiotoxicity caused by tricyclic antidepressants and is commonly recommended in flecainide toxicity, but direct evidence in pilsicainide intoxication is limited [10,11]. Its potential benefits must be balanced against hypernatremia, alkalemia, and sodium and volume loading, particularly in patients with pulmonary congestion or advanced kidney dysfunction.

Several case reports describe clinical and electrocardiographic recovery during hemodialysis or CHDF [3-5,7]. Horita et al. reported shortening of electrocardiographic intervals in parallel with declining serum pilsicainide concentrations during fluid administration and CHDF [4]. Yasuda et al. described polymorphic ventricular tachycardia that persisted after correction of hyperkalemia and resolved during CHDF while the serum pilsicainide concentration declined, although it remained above the reported therapeutic range when sinus rhythm returned [7]. Asano et al. reported resolution of QRS prolongation and neuropsychiatric manifestations during continuous blood purification [3]. Hirano et al. reported disappearance of accelerated idioventricular rhythm after emergency hemodialysis [5]. These reports support considering renal replacement therapy in severe toxicity, particularly when renal failure, electrolyte abnormalities, or volume overload provide an independent indication. They do not establish the isolated efficacy of dialysis because drug withdrawal and correction of metabolic abnormalities occurred simultaneously, and reported dialytic removal of pilsicainide is limited. In our patient, although hemodynamics were preserved at presentation with a blood pressure of 156/102 mmHg, CHDF was selected rather than intermittent hemodialysis. This decision was guided by the need for gradual and continuous fluid removal to manage pulmonary congestion and peripheral edema, as well as concern for potential hemodynamic collapse secondary to profound conduction delay and marked bradycardia from severe pilsicainide cardiotoxicity.

Comparison with other sodium channel blocker toxicities

Flecainide toxicity also causes marked conduction slowing and wide QRS rhythms that may be mistaken for ventricular tachycardia [4]. However, pharmacokinetic and therapeutic observations from flecainide cannot be directly extrapolated to pilsicainide. In either setting, recognition of sodium channel blocker toxicity is important because additional class I sodium channel-blocking antiarrhythmic therapy may further impair conduction. Treatment should be individualized according to the rhythm, hemodynamic status, electrolyte and acid-base abnormalities, and pharmacokinetic characteristics of the causative drug.

## Conclusions

Pilsicainide intoxication should be suspected when patients with impaired renal function present with unexplained bradyarrhythmias or relatively slow, regular, wide QRS rhythms. In the present case, acute-on-chronic renal deterioration provided a plausible mechanism for severe pilsicainide accumulation, which likely contributed to the emergence of alternating junctional escape rhythm and an accelerated idioventricular rhythm-like wide QRS pattern with marked intraventricular conduction slowing.

Differentiating this toxidrome from ventricular tachycardia relies on comprehensive clinical evaluation - incorporating exposure history, serial renal and electrolyte profiles, serum drug levels, and longitudinal electrocardiographic changes - rather than isolated criteria. Timely drug cessation alongside multi-faceted metabolic and volume management, including continuous renal replacement therapy when indicated, was associated with favorable clinical and electrophysiological resolution.
